# Prognostic Nutritional Index and Systemic Immune-Inflammation Index Predict the Prognosis of Patients with HCC

**DOI:** 10.1007/s11605-019-04492-7

**Published:** 2020-02-05

**Authors:** Dong Wang, Xi Hu, Liang Xiao, Guo Long, Lei Yao, ZhiMing Wang, LeDu Zhou

**Affiliations:** Department of General Surgery, Xiangya Hospital, Central South University, 87 Xiangya Road, Changsha, 410008 Hunan China

**Keywords:** Prognostic nutritional index, Systemic immune-inflammation index, Hepatocellular carcinoma

## Abstract

**Introduction:**

Systemic nutrition and inflammation are the critical factors in cancer initiation, evolution, and progression. This study aimed to evaluate the prognostic value of the prognostic nutritional index (PNI) and systemic immune-inflammation index (SII) in hepatocellular carcinoma (HCC) patients who underwent liver resection.

**Methods:**

A total of 202 HCC patients met the criteria and were included in the study. The receiver operating characteristic (ROC) curve was used to calculate the optimal PNI and SII cutoff values. The relationship between PNI/SII and clinicopathologic parameters was analyzed. The effect of PNI and SII on recurrence-free survival (RFS) and overall survival (OS) was investigated by Kaplan-Meier curves and Cox proportional hazards models.

**Results:**

The areas under the ROC curve for PNI and SII were 0.64 and 0.58. The ideal preoperative PNI and SII cutoff values were 50.25 and 461.5, respectively. Multivariate Cox regression analysis identified that the PNI (*P* = 0.001) and tumor diameter (*P* = 0.018) were significant prognostic markers for RFS, and that the PNI (*P* = 0.049), SII (*P* = 0.039) and tumor diameter (*P* = 0.001) were significant prognostic markers for OS. The median RFS in the PNI-low and PNI-high groups was 13.5 months and 23 months (*P* = 0.001), and that in the SII-low and SII-high groups was 18 months and 15 months (*P* = 0.03), respectively. The median OS in the PNI-low and PNI-high groups was 24 months and 39 months (*P* = 0.001), and that in the SII-low and SII-high groups was 36 months and 22 months (*P* = 0.002), respectively.

**Conclusion:**

Interestingly, we found that PNI and SII could be important prognostic parameters for HCC patients who under hepatectomy.

## Introduction

Hepatocellular carcinoma (HCC) is one of the most common malignant tumors of the digestive system and has high morbidity and mortality worldwide. In China, liver cancer is the fourth highest incidence rate and the third highest mortality rate.^1^ In the USA, the incidence of liver cancer is rising faster than that for any other cancers, and the 5-year relative survival rate is only 18%.^2^ The burden of HCC is increasing and might surpass an annual incidence of 1 million cases.^3^ HCC most commonly occurs with hepatitis infection (including hepatitis B virus (HBV), HCV), alcohol abuse, and non-alcoholic fatty liver disease. Cirrhosis underlines most cases of HCC and is an independent risk factor for HCC. Liver resection, liver transplantation, and radiofrequency ablation are the first-line radical treatments for HCC.^4, 5^ For HCC patients, especially those with advanced-stage HCC, who are not candidates for radical treatments, locoregional therapies,^6^ targeted therapies,^3^ and immunotherapy^7^ are the most widely used treatments. Researchers reported a 5-year overall survival (OS) rate of 50–70% for HCC patients, but the high recurrence rate following curative surgery limits the treatment outcome.^8, 9^ The traditional prognostic parameters of HCC patients, including tumor size, vascular invasion, and other factors obtained after surgical resection, are considered inconveniently in daily clinical work. Accordingly, finding novel prognostic parameters to help predict the clinical outcomes of HCC patients is warranted.

Inflammation is considered one of the enabling characteristics in tumorigenesis, progression, and metastasis.^10^ Chronic inflammation is associated with an elevated risk of cancers;^11^ for example, HBV infection leads to HCC, EBV leads to nasopharyngeal carcinoma, and *Helicobacter pylori* leads to gastric cancer. With the increasing understanding of cancer-related inflammation, systemic inflammatory response biomarkers, such as the neutrophil-to-lymphocyte ratio, platelet-to-lymphocyte ratio, lymphocyte-to-monocyte ratio, and C-reactive protein level,^12^ have been clearly demonstrated to predict the prognoses of cancer patients, including those with non-small cell lung cancer,^13^ renal cell carcinoma,^14^ and colorectal cancer.^15^ However, inflammation prognosis biomarkers only integrate two inflammatory cells. The systemic immune-inflammation index (SII) is calculated by platelet, neutrophil, and lymphocyte counts and is a novel indicator that can predict the clinical outcomes of cancer patients.^16–18^

The status of nutrition is also closely associated with postoperative complications and the clinical outcomes of patients with various solid tumors. Recently, many indicators containing nutritional and inflammatory variables have been found to play a role in predicting the prognoses for cancer patients, such as those with colorectal cancer,^19^ esophageal cancer,^20^ and so on. The concept of the prognostic nutritional index (PNI) was first proposed by Buzby et al.^21^ to evaluate the risk of gastrointestinal surgery. The PNI, which is calculated based on the serum albumin and circulating peripheral blood lymphocyte count, has been used to assess the immunonutritional status of cancer patients. The PNI has also been verified as a useful prognostic biomarker in various cancers, including esophageal carcinoma and osteosarcoma.^22, 23^ In HCC patients, the albumin is also regarded as the important factor for the liver function.

Although increasing evidence shows that the SII and PNI, which can reflect systemic inflammation and nutritional statuses, can accurately predict cancer patient prognosis, the relationship between PNI, SII, and clinical outcomes in patients with HCC remains unclear and has not been verified. The main purpose of this study was to evaluate the prognostic value of SII and PNI in HCC patients who underwent surgical resection.

## Methods and Materials

### Study Population and Design

In our study, 202 HCC patients were included, and all patients underwent hepatectomy between 2012 and 2015 at Xiangya Hospital, Central South University. After surgical resection, all patients were diagnosed with HCC by pathology. Patients who met any one of the following criteria were excluded from this study: (1) patients with previous splenectomy; (2) patients with tumor rupture and bleeding; (3) patients with recurrence HCC; (4) patients with infectious diseases before hepatectomy, such as lung infections and urinary tract infections; (5) patients with autoimmune diseases; (6) patients who underwent antitumor therapies; and (7) patients with missing clinical data. This study was approved by the ethics committee of Xiangya Hospital, Central South University, and the patients provided informed consent.

### Follow-up and Definitions

All HCC patients underwent preoperative blood routine and liver function tests before surgical resection, and the PNI was calculated as albumin level (g/L) + 5× total lymphocyte count (10^9^/L). The SII was defined as platelet × neutrophil/lymphocyte counts. The clinicopathological data of the HCC patients including sex, age, HBV infection, tumor size, tumor number, tumor-node-metastasis (TNM) stage, and so on.

Serum alpha-fetoprotein (AFP), prothrombin time (PT), activated partial thromboplastin time (APTT), white blood cell (WBC) count, platelet (PLT) count, alanine transaminase (ALT), aspartate transaminase (AST), and hepatitis B surface antigen (HBsAg) were tested in all patients. Abdominal ultrasonography, computed tomography (CT), magnetic resonance imaging (MRI), and chest radiography were performed for all patients. The PNI and SII cutoff values were calculated by receiver operating characteristic (ROC) curves according to the OS of patients. In our study, tumor number within the liver was divided into two groups: the single tumor group and the multiple tumors group. The seventh edition of the American Joint Committee on Cancer TNM staging system and Barcelona Clinic Liver Cancer (BCLC) scores were applied to classify the HCC stage.

### Statistical Analysis

Statistical analysis was performed using Prism software (GraphPad Prism Software, La Jolla, CA) and SPSS 21.0 (SPSS Company, Chicago, IL) for Windows. Student’s *t* tests were used to analyze the quantitative data. Chi-square tests or Fisher’s exact tests were used to analyze the categorical variables. Spearman’s correlation was performed to analyze the correlations between PNI/SII and other clinicopathological parameters. Recurrence-free survival (RFS) and OS were evaluated using the Kaplan-Meier method. Cox proportional hazards regression was used to determine the prognostic factors associated with OS and RFS with univariate and multivariate analyses. The hazard ratio (HR) and 95% confidence interval (CI) were used to describe the relative risk factors. A *P* value < 0.05 was considered statistically significant.

## Results

### Patients and Tumor Characteristics

In this study, 202 HCC patients who met the inclusion criteria were enrolled.

There were 168 males and 34 females, and the average age was 50.41 ± 11.95 years. The HBV infection proportion was 83.66%, and 24.26% of the patients had multiple tumors. The median serum albumin level was 41.39 ± 3.75 g/L. The average neutrophil, lymphocyte, and plate counts were (3.46 ± 1.35)10^9^/L, (1.49 ± 0.54)10^9^/L, and (161.72 ± 69.06)10^9^/L, respectively. The mean ALT and AST were 43.28 ± 34.19 U/L and 46.90 ± 32.98 U/L, respectively; the mean total bilirubin and direct bilirubin were 14.94 ± 12.0 μmol/L and 5.94 ± 6.55 μmol/L, respectively. The mean tumor diameter was 5.77 ± 3.38 cm. The mean PNI and SII were 48.83 ± 4.8 and 433.23 ± 325.61, respectively. The proportion of patients with BCLC stages of 0/A, B, and C were 69.80%, 19.80%, and 10.40%, respectively (Table 1).

**Table 1:** The clinicopathological variables in patients with HCC (*n* = 202)

Variables	Values (*n* = 202)
Age (years), mean (SD)	50.41 (11.95)
Albumin (g/L), mean (SD)	41.39 (3.75)
WBC (10^9^/L), mean (SD)	5.63 (1.67)
Neutrophil (10^9^/L), mean (SD)	3.46 (1.35)
Lymphocyte (10^9^/L), mean (SD)	1.49 (0.54)
Monocyte (10^9^/L), mean (SD)	0.48 (0.2)
PLT (10^9^/L), mean (SD)	161.72 (69.06)
Total bilirubin (μmol/L), mean (SD)	14.94 (12)
Direct bilirubin (μmol/L), mean (SD)	5.94 (6.55)
ALT (U/L), mean (SD)	43.28 (34.19)
AST (U/L), mean (SD)	46.90 (32.98)
PT (s), mean (SD)	13.2 (0.98)
APTT (s), mean (SD)	33.11 (4.32)
Tumor diameter (cm), mean (SD)	5.77 (3.38)
Creatinine (μmol/L), mean (SD)	86.55 (16.5)
PNI	48.83 (4.8)
SII	433.23 (325.61)
Hospital stay (days), mean (SD)	15.43 (4.33)
Gender	
Male	168
Female	34
HBV	
Absence	33
Presence	169
Tumor capsular	
Absence	142
Presence	60
AFP	
≤ 20 ng/mL	74
> 20 ng/mL	128
Edmondson grade	
I–II	152
III–IV	50
Tumor number	
Single	153
Multiple	49
Satellite nodules	
Absence	186
Presence	16
Cirrhosis	
Absence	52
Presence	152
BCLC stage	
0/A	141
B	40
C	21
TNM stage	
I/II	167
III	35

*WBC* white blood cell, *PLT* platelet, *ALT* alanine transaminase, *AST* aspartate transaminase, *PT* prothrombin time, *PNI* prognostic nutritional index, *SII* systemic immune-inflammation index, *AFP* alpha-fetoprotein, *BCLC* Barcelona Clinic Liver Cancer, *TNM* tumor-node-metastasis

### Assessment of the PNI and SII Cutoff Values

**Fig. 1:**
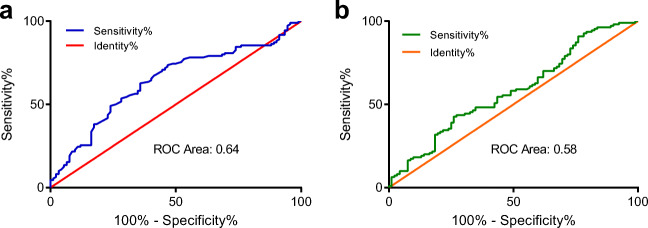
The ROC curves of PNI and SII in HCC patients. **a** The ROC curve for PNI. The area under the ROC curve was 0.64 (95% CI = 0.56–0.72; *P* < 0.05), and the optimal cutoff value was 50.25. **b** The ROC curve for SII. The area under the ROC curve was 0.58 (95% CI = 0.50–0.66; *P* < 0.05), and the optimal cutoff value was 461.5

*The optimal PNI and SII cutoff values were analyzed by ROC curves according to the OS of patients with HCC*. According to the ROC curve and the Youden index, the ideal preoperative PNI and SII cutoff values were 50.25 and 461.5, respectively. The areas under the ROC curve for PNI and SII were 0.64 (95% CI for the area between 0.56 and 0.72, *P* < 0.05) (Fig. 1a) and 0.58 (95% CI for the area between 0.50 and 0.66, *P* < 0.05) (Fig. 1b), respectively. The PNI and SII cutoff values correspond to sensitivity values of 73.64% and 42.73% and specificity values of 53.26% and 73.91%, respectively. For subsequent data analysis, the patients were divided into two groups for further analysis based on the ideal cutoff values: the PNI-low group (PNI ≤ 50.25) and PNI-high group (PNI > 50.25), and the SII-low group (SII ≤ 461.5) and SII-high group (SII > 461.5).

### Univariate and Multivariate Analyses for the HCC Patients

Univariate and multivariate analyses were performed for age, serum albumin, ALT, AST, tumor diameter, and other clinicopathologic variables. The univariate Cox regression analysis identified that serum albumin (HR = 0.87; 95% CI = 0.78–0.97; *P* = 0.013), tumor diameter (HR = 1.29; 95% CI = 1.09–1.53; *P* = 0.004), and PNI (HR = 0.17; 95% CI = 0.07–0.39; *P* = 0.01) were significant prognostic factors associated with RFS. Serum albumin (HR = 0.88; 95% CI = 0.81–0.95; *P* = 0.001), tumor diameter (HR = 1.19; 95% CI = 1.08–1.32; *P* = 0.001), PNI (HR = 0.31; 95% CI = 0.17–0.57; *P* = 0.02), and SII (HR = 2.08; 95% CI = 1.15–3.76; *P* = 0.016) were significant prognostic factors associated with OS (Table 2).

**Table 2:** Univariate and multivariate analyses for RFS and OS in patients with HCC

Variables	RFS		OS	
	HR (95% CI)	*P* value	HR (95% CI)	*P* value
Univariate analysis				
Age (years)	1.01 (0.98–1.04)	0.516	0.99 (0.97–1.02)	0.537
Albumin (g/L)	0.87 (0.78–0.97)	0.013	0.88 (0.81–0.95)	0.001
WBC (10^9^/L), mean (SD)	0.84 (0.68–1.04)	0.115	1.09 (0.93–1.30)	0.295
Neutrophil (10^9^/L), mean (SD)	0.82 (0.63–1.07)	0.138	1.14 (0.92–1.40)	0.23
Lymphocyte (10^9^/L), mean (SD)	0.63 (0.32–1.22)	0.168	0.75 (0.44–1.26)	0.274
Monocyte (10^9^/L), mean (SD)	0.59 (0.10–3.45)	0.559	3.91 (0.90–17.08)	0.069
PLT (10^9^/L)	1.00 (1.00–1.01)	0.54	1.00 (1.00–1.01)	0.189
Total bilirubin (μmol/L)	0.99 (0.97–1.01)	0.394	1.00 (0.97–1.02)	0.734
Direct bilirubin (μmol/L)	0.98 (0.94–1.03)	0.411	0.99 (0.95–1.04)	0.672
ALT (U/L)	1.01 (0.99–1.03)	0.24	1.00 (0.99–1.01)	0.923
AST (U/L)	1.00 (0.99–1.01)	0.794	1.00 (0.99–1.00)	0.291
APTT (s)	1.01 (0.92–1.11)	0.796	0.97 (0.91–1.03)	0.313
PT (s)	1.02 (0.69–1.51)	0.925	0.97 (0.73–1.29)	0.843
Tumor diameter (cm)	1.29 (1.09–1.53)	0.004	1.19 (1.08–1.32)	0.001
Creatinine (μmol/L)	0.99 (0.97–1.01)	0.352	1.00 (0.98–1.01)	0.534
HBV (presence vs. absence)	1.99 (0.57–6.96)	0.284	1.00 (0.48–2.12)	0.991
Tumor capsular (absence vs. presence)	1.04 (0.45–2.41)	0.929	1.07 (0.58–1.95)	0.835
AFP (ng/mL) (> 20 vs. ≤ )	1.06 (0.48–2.36)	0.885	1.50 (0.84–2.69)	0.169
Edmondson grade (III–IV vs. I–II)	1.86 (0.67–5.13)	0.232	1.51 (0.78–2.90)	0.218
Tumor number (multiple vs. single)	3.44 (1.00–11.84)	0.051	1.61 (0.83–3.13)	0.157
PNI (high group vs. low group)	0.17 (0.07–0.39)	0.01	0.31 (0.17–0.57)	0.02
SII (high group vs. low group)	1.23 (0.54–2.77)	0.625	2.08 (1.15–3.76)	0.016
Sex (male vs. female)	3.34 (0.76–14.72)	0.111	1.95 (0.89–4.25)	0.094
Cirrhosis (presence vs. absence)	1.46 (0.64–3.35)	0.369	1.19 (0.63–2.26)	0.587
TNM stage (III vs. I/II)	2.40 (0.69–8.36)	0.169	1.79 (0.86–3.73)	0.122
Multivariate analysis				
Albumin (g/L)	1.07 (0.92–1.25)	0.377	0.98 (0.88–1.09)	0.705
PNI (high group vs. low group)	0.11 (0.03–0.39)	0.001	0.43 (0.19–1.00)	0.049
SII (high group vs. low group)	1.14 (0.46–2.87)	0.776	1.98 (1.04–3.77)	0.039
Tumor number (multiple vs. single)	2.41 (0.64–9.20)	0.194		
Tumor diameter (cm)	1.27 (1.04–1.54)	0.018	1.20 (1.08–1.33)	0.001

*WBC* white blood cell, *PLT* platelet, *ALT* alanine transaminase, *AST* aspartate transaminase, *PT* prothrombin time, *PNI* prognostic nutritional index, *SII* systemic immune-inflammation index, *AFP* alpha-fetoprotein, *TNM* tumor-node-metastasis

From the multivariate analyses, we found that PNI (HR = 0.11; 95% CI = 0.03–0.39; *P* = 0.001) and tumor diameter (HR = 1.27; 95% CI = 1.04–1.54; *P* = 0.018) were significant prognostic markers for RFS, and that PNI (HR = 0.43; 95% CI = 0.19–1.00; *P* = 0.049), SII (HR = 1.98; 95% CI = 1.04–3.77; *P* = 0.039), and tumor diameter (HR = 1.20; 95% CI = 1.08–1.33; *P* = 0.001) were significant prognostic markers for OS (Table 2).

### The Prognostic Value of PNI and SII in HCC Patients

**Fig. 2:**
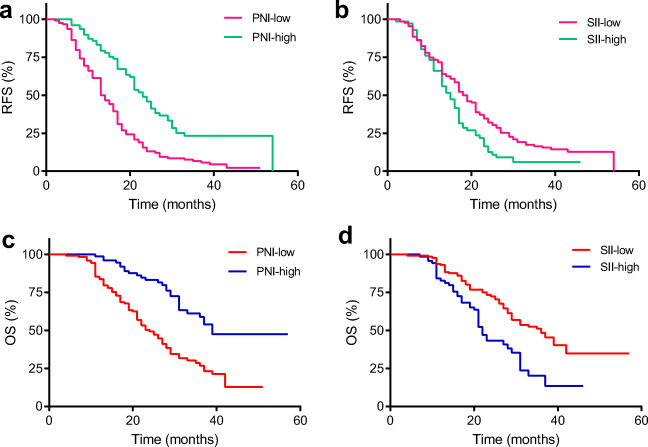
The Kaplan-Meier survival curves for RFS and OS in HCC patients. **a** The median RFS times in the PNI-low and PNI-high groups were 13.5 months and 23 months, respectively (*P* = 0.001). **b** The median RFS times in the SII-low and SII-high groups were 18 months and 15 months (*P* = 0.03), respectively. **c** The median OS times in the PNI-low and PNI-high groups were 24 months and 39 months, respectively (*P* = 0.001). **d** The median OS times in the SII-low and SII-high groups was 36 months and 22 months, respectively (*P* = 0.002)

Finally, we evaluated the prognostic value of PNI and SII in HCC patients who underwent liver resection. The median RFS times in the PNI-low and PNI-high groups were 13.5 months and 23 months (*P* = 0.001, Fig. 2a), respectively, and the median RFS times in the SII-low and SII-high groups were 18 months and 15 months (*P* = 0.03, Fig. 2b), respectively. The median OS times in the PNI-low and PNI-high groups were 24 months and 39 months (*P* = 0.001, Fig. 2c), respectively, and the median OS in the SII-low and SII-high groups was 36 months and 22 months (*P* = 0.002, Fig. 2d), respectively.

## Discussion

A lot of research studies have found that the PNI and SII play an important role in the cancer development and prognosis. In our study, we divided the patients into two group: PNI(SII) high group and PNI(SII) low group. And, we interestingly found that PNI and SII can predict the HCC prognosis in patients who had underwent liver resection, and PNI and SII were independent risk factors for the OS of HCC patients.

Inflammation is closely related to the occurrence and metastasis of tumors, and the immune and nutritional status of the system is an important part of the inflammatory response. Therefore, an increasing number of studies have focused on the role of inflammation and nutrition in cancer patients, including those with pancreatic cancer^24^ and osteosarcoma.^23^ Many studies have reported that PNI and SII have great clinical significance in evaluating the prognosis in many solid cancers.

The PNI is calculated based on lymphocyte count and serum albumin levels. By releasing TNF, interferon-γ, and other cytokines, lymphocytes can restrain cancer cell growth and metastasis. The reduction in lymphocyte count can weaken bodily immune functions, and cancer cells are more prone to immune escape and lead to poor prognosis in cancer patients. Ostroumov et al.^25^ reported that CD4 and CD8 T lymphocytes can mediate the growth of cancer cells. Serum albumin is produced by the liver and is an important nutritional index of the body. And in the HCC patients who underwent liver resection, the lower albumin level means the HCCs may have had liver dysfunction even liver failure which had high mortality rate and worse clinical outcomes.

The reasons why PNI can predict the prognosis of cancer patients are as follows: (1) lymphocytes mainly participate in the immune response and inhibit tumor cell proliferation and metastasis. A lower lymphocyte count can weaken the systemic immune system, and the cancer cells easily escape from immune surveillance and ultimately enhance the malignant biological behavior of cancer cells. (2) Serum albumin is the most simple and effective parameter for reflecting the body’s nutritional status, which is a decisive factor in cancer cell immune reactions. Moreover, serum albumin can be used to evaluate liver function in HCC patients. Hypoalbuminemia downregulates the systemic immune system and leads to tumor cell proliferation. Therefore, lymphocytes combined with serum albumin can predict the prognosis of cancer patients.

SII is an accurate systemic inflammatory index based on neutrophil, lymphocyte, and platelet counts. SII can accurately reflect the degree of systemic inflammation. Moreover, inflammation plays an important role in the tumor microenvironment. Many inflammatory cells and cytokines in the tumor microenvironment can affect cancer occurrence, development, and metastasis. A growing number of studies have demonstrated that platelets, neutrophils, and lymphocytes, can influence the biological behavior of cancer cells,^26–29^ including aggregate in vessels and release of vascular endothelial growth factor, TGF-beta, platelet-derived growth factor, etc., which can promote the differentiation of tumor cells and tumor proliferation. In addition, cancer cells can release thrombopoietin and inflammatory mediators, which can promote platelet growth, in turn promoting tumor growth. Neutrophils can promote angiogenesis, tumor growth, and metastasis by releasing VEGF and matrix metalloproteinase. Many studies have shown that extensive neutrophil infiltration in tumor tissues is associated with cancer recurrence and is an independent risk factor for poor prognosis in cancer patients. In this study, we found that SII was negatively correlated with albumin and AST, indicating that SII can also damage the liver function in HCC patients. A higher SII score in HCC patients usually indicated poorly liver function, which is a negative risk factor for the clinical outcomes of these patients.

In addition, PNI and SII are simple, practical, and effective biomarkers for the routine examination of patients with tumors because these indices can be evaluated by routine blood and liver function tests. There were also limitations in our study: firstly, our research was a retrospective study, and the sample size was not large. Therefore, multicenter, large-sample clinical studies are needed to obtain more accurate PNI and SII value and to accurately predict the prognosis of cancer patients. Secondly, the optimal PNI and SII values vary in different studies because of diverse sample sizes and patient selection criteria, resulting in bias in the values. Thirdly, there were many HBV-related HCC patients in our study, but we can analyze the viral load which may alter the PNI/SII. For patients who had poor prognosis by the PNI and SII, early treatment such as TACE and targeted therapy may improve the prognosis and prolong the survival time in HCC patients.

## Conclusion

PNI and SII can predict the prognosis of HCC patients who underwent hepatectomy.

## Abbreviations

AFP: Alpha-fetoprotein
ALT: Alanine transaminase
AST: Aspartate transaminase
BCLC: Barcelona Clinic Liver Cancer
HCC: Hepatocellular carcinoma
PLT: Platelet
PNI: Prognostic nutritional index
PT: Prothrombin time
SII: Systemic immune-inflammation index
TNM: Tumor-node-metastasis
WBC: White blood cell
